# Primary pulmonary myxoid sarcoma with EWSR1::CREB1 fusion: a literature review

**DOI:** 10.1007/s00432-024-05634-4

**Published:** 2024-02-29

**Authors:** Xinyu Miao, Jing Chen, Lan Yang, Hongyang Lu

**Affiliations:** 1 The Second Clinical Medical College, Zhejiang Chinese Medical University, Hangzhou, 310053 People’s Republic of China; 2 Zhejiang Key Laboratory of Diagnosis and Treatment Technology On Thoracic Oncology (Lung and Esophagus), Zhejiang Cancer Hospital, Hangzhou Institute of Medicine (HIM), Chinese Academy of Sciences, Hangzhou, 310022 People’s Republic of China; 3 Department of Thoracic Medical Oncology, Zhejiang Cancer Hospital, Hangzhou Institute of Medicine (HIM), Chinese Academy of Sciences, Hangzhou, 310022 People’s Republic of China; 4 Postgraduate Training Base Alliance of Wenzhou Medical University (Zhejiang Cancer Hospital), Hangzhou, Zhejiang 310022 People’s Republic of China

**Keywords:** PPMS, *EWSR1::CREB1* fusion, Treatment, Prognosis

## Abstract

**Purpose:**

This review primarily aims to review the epidemiology, clinical characteristics, imaging, pathology, immunohistochemistry, diagnosis, differential diagnosis, treatment, and prognosis of Primary pulmonary myxoid sarcoma (PPMS) with EWS RNA binding protein 1::cAMP response element binding protein 1 (EWSR1::CREB1) fusion. It provides reference for the diagnosis and treatment of this disease.

**Methods:**

Retrospectively collected the literature about PPMS with EWSR1::CREB1 fusion, its clinical, radiology, histology, molecular characteristics and current treatment strategies were collated and analyzed. This review provides a detailed differential diagnosis of the disease.

**Results:**

PPMS is an exceptionally rare, low-grade malignant tumor of the lung. This tumor commonly infiltrates lung tissue and develops within bronchial passages. It is identified by a genetic rearrangement involving the EWSR1 gene and a distinct chromosomal translocation t(2; 22)(q33; q12). Variants include EWSR1::CREB1 fusion and EWS RNA binding protein 1::activating transcription factors (EWSR1::ATF1) fusion. PPMS with EWSR1::CREB1 fusion is more prevalent among middle-aged individuals and affects both sexes almost equally. Clinical symptoms are relatively non-specific, primarily including cough, hemoptysis, and weight loss. Most patients undergo surgery and experience a favorable prognosis. Further research is required to validate the effectiveness of alternative treatments for PPMS with EWSR1::CREB1 fusion.

**Conclusion:**

EWSR1 rearrangement and EWSR1::CREB1 fusion are crucial genetic features of PPMS and serve as important diagnostic markers. Immunohistochemically, PPMS tests positive for EMA. In terms of treatment, surgery has been the primary approach in recent years. Therefore, the efficacy of other treatments still requires further investigation.

## Introduction

Primary pulmonary myxoid sarcoma (PPMS) is an exceedingly rare, low-grade malignant sarcoma. It was initially described as a primary pulmonary endobronchial myxoid tumor in the study of Nicholson AG et al. in 1999 (Nicholson et al. 1999). PPMS stands out due to its distinctive morphological and genetic traits, notably the presence of the EWS RNA binding protein 1::cAMP response element binding protein 1 (*EWSR1::CREB1*) fusion gene and a remarkably low Ki-67 proliferation index (Wu et al. 2021). Thway K and associates further uncovered the relationship between characteristic chromosomal translocation t(2; 22) (q33; q12) and the occurrence of *EWSR1::CREB1* fusion (Thway et al. 2011). PPMS was first recognized by World Health Organization (WHO) classification in 2015, which belonged to the category of mesenchymal tumors. Then it was classified as mesenchymal tumors specific to the lung in the 2021 WHO Classification of Lung Tumors (Travis et al. 2015; Nicholson et al. 2022). PPMS originates from mesenchymal components in the bronchial wall, pulmonary stroma, or blood vessels (Gołota et al. 2019). Its clinical presentation is generally unspecific, sometimes detectable only through physical examination, making it challenging to differentiate from other lung tumors. This article primarily aims to review the epidemiology, clinical characteristics, imaging, pathology, immunohistochemistry, diagnosis, differential diagnosis, treatment, and prognosis of PPMS with *EWSR1::CREB1* fusion.

### Epidemiology and clinical characteristics

PPMS is an exceptionally rare, low-grade sarcoma, with a total of 39 clinical cases compiled in Table 1 (Nicholson et al. 1999; Wu et al. 2021; Thway et al. 2011; Matsukuma et al. 2012; Chen et al. 2020; Inayama et al. 2001; Smith et al. 2014; Jeon et al. 2014; Kim et al. 2017; Yanagida et al. 2017; Agaimy et al. 2017; Koelsche et al. 2020; Opitz et al. 2019; Nishimura et al. 2023; Prieto-Granada et al. 2017). Most of the symptoms of PPMS that manifest clinically are non-specific, such as cough, hemoptysis, and weight loss. Among the 39 cases, 20 were in female patients and 19 in male patients, resulting in a male-to-female ratio of 0.95:1. The age range of PPMS cases spanned from 21 to 80 years, with a median age of 43.6. Among the documented cases, 12 individuals had never smoked, while 11 had a history of smoking. This suggests that the occurrence of PPMS is not directly linked to smoking history. PPMS does not display a specific predilection for a particular location within the lung; it can invade both the right and left lungs, though it is more frequently found in the right lung (59%, 23/39). PPMS commonly infiltrates bronchial tissues, with a tendency toward endobronchial growth observed in 17 out of 39 cases, while 4 cases exhibited no endobronchial involvement, and the remaining cases did not provide sufficient information. Nonetheless, this tumor can also develop outside the lung parenchyma, occurring within an interlobar fissure without parenchymal invasion (Kim et al. 2017).

**Table 1:** Clinical, histologic, and pathological features of PPMS

No	age	sex	smoking history	EBC (±)	site	Tumor size (cm)	Histologic and pathological features	Mitosis	References
1	27	F	No	EBC +	RLL	4	Ill-defined nodules of myxoid stroma, which contained interweaving cords of small uniform, rounded or slightly elongated cells with small volumes of PAS-positive eosinophilic cytoplasm. The nodules were interspersed by fibrous septa of varying thickness. The myxoid stroma stained positively with Alcian blue, focal hemosiderin deposition	Occasional mitoses were seen	Nicholson et al. (1999)
2	43	F	Yes	EBC +	L	13	Ill-defined nodules of myxoid stroma, which contained interweaving cords of small uniform, rounded or slightly elongated cells with small volumes of PAS-positive eosinophilic cytoplasm. The nodules were interspersed by fibrous septa of varying thickness. The myxoid stroma stained positively with Alcian blue, focal hemosiderin deposition	Occasional mitoses were seen	Nicholson et al. (1999)
3	24	M	No	NM	RLL	5	NM	NM	Wu et al. (2021)
4	64	F	No	NM	RUL	5.5	NM	NM	Wu et al. (2021)
5	27	M	Yes	NM	RLL	5	NM	NM	Wu et al. (2021)
6	45	M	Yes	NM	LLL	< 3	NM	NM	Wu et al. (2021)
7	43	M	Yes	NM	RLL	2	NM	NM	Wu et al. (2021)
8	23	M	Yes	NM	RLL	< 3	NM	NM	Wu et al. (2021)
9	45	F	No	NM	RUL	2	NM	NM	Wu et al. (2021)
10	49	M	Yes	NM	R	14	NM	NM	Wu et al. (2021)
11	47	M	NM	NM	RUL	2	NM	NM	Wu et al. (2021)
12	44	M	No	NM	LUL	2	Spindle cell proliferative lesions, with rich mucoid background and accompanied by a large lymphocyte and plasma cell infiltrate. The tumor cells were distributed in a nodular manner with significant collagen interstitium. Tumor cells were round and oval and arranged in a reticular or ribbon-like arrangement in a mucoid background. Atypia in tumor cells was mild to moderate	The mitotic activity was lower than 2/10 HPF (high-power fields)	Wu et al. (2021)
13	27	F	Yes	EBC +	RLL	4	Well-circumscribed, lobulated, reticular network with delicate lace-like strands and cords of cells within prominent myxoid stroma, tumor cells showed no or minimal atypia	NM	Thway et al. (2011)
14	33	F	Yes	EBC +	LUL	3.5	Lobulated, the tumor cells were distributed in a reticular manner. Atypia in tumor cells was mild to moderate	NM	Thway et al. (2011)
15	45	F	NM	EBC +	RUL	1.5	Circumscribed, lobulated, with peripheral fibrosis. Atypia in tumor cells was mild to moderate	NM	Thway et al. (2011)
16	36	F	NM	NM	L	NM	Circumscribed, with fibrous pseudocapsule, the tumor cells were distributed in a reticular manner	NM	Thway et al. (2011)
17	32	F	NM	EBC +	RUL	NM	Lobulated, the tumor cells were distributed in a reticular manner. Atypia in tumor cells was moderate	NM	Thway et al. (2011)
18	28	M	No	EBC +	LLL	2.8	Infiltrative and partially lobulated. Atypia in tumor cells was mild to moderate	NM	Thway et al. (2011)
19	67	M	Yes	EBC +	LLL	2.8	Well circumscribed and lobulated, the tumor cells were distributed in a reticular manner	NM	Thway et al. (2011)
20	68	F	NM	EBC +	RUL	2	Well circumscribed and lobulated, the tumor cells were distributed in a reticular manner. Atypia in tumor cells was moderate to focally marked	NM	Thway et al. (2011)
21	63	F	Yes	EBC +	LUL	NM	Lobulated, the tumor cells were distributed in a reticular manner	NM	Thway et al. (2011)
22	51	M	NM	NM	RLL	2	Well circumscribed and lobulated, the tumor cells were distributed in a reticular manner. Atypia in tumor cells was moderate to focally marked	NM	Thway et al. (2011)
23	31	M	No	EBC +	LLL	2.7	Well-circumscribed, multinodular arrangement and be composed of reticular cords of oval, short spindle, or polygonal cells with swollen vesicular nuclei containing occasional vacuolar inclusions, accompanied by an abundant myxoid stroma and scattered lymphoplasmacytic infiltrates	Rare mitotic figures	Matsukuma et al. (2012)
24	45	F	No	EBC-	RUL	2.1	Well-circumscribed, multinodular, reticular network of delicate lace-like cellular strands and cords in abundant myxoid stroma, chondrocyte or physaliferous-like tumor cells with mild atypia	Rare mitotic figures	Chen et al. (2020)
25	60	M	NM	NM	R	9	Well circumscribed and showed gelatinous. Composed of slightly atypical, spindle-shaped, or stellate cells, which were loosely distributed within a prominent myxoid stroma. The myxoid ground substance was composed of hyaluronic acid and acid mucopolysaccharide	Rare mitotic figures	Inayama et al. (2001)
26	66	F	NM	EBC +	LUL	4	Polygonal to spindled cells with a reticular, myoepithelial-type appearance and mild atypia in a pink–gray myxoid stroma. A peritumoral fibrous cuff was present, particularly at the interface with alveolar parenchyma	NM	Smith et al. (2014)
27	28	M	NM	NM	RLL	8.5	Lobular, biphasic lesion, ~ 40% of which was composed of myxoid pools. The remainder showed exuberant fibroinflammatory reaction with confluent plasma cells. Two foci showed "angiomatoid" blood-filled spaces. Moderate atypia was present among reticular and clustered cells floating in the distinctive EMC-type stroma	NM	Smith et al. (2014)
28	28	M	NM	EBC +	RUL	6	At low magnification, the tumor showed a destructive growth pattern, with focal necrosis and infiltration from and endobronchial base into adjacent lung parenchyma. At higher power, the degree of atypia was severe, and the myxoinflammatory areas expanded to undermine the bronchial epithelium	NM	Smith et al. (2014)
29	26	M	NM	EBC +	LLL	9	Well circumscribed, multinodular, and distinctly gelatinous, consisted of vague coalescent nodules composed of short spindle to ovoid or stellate cells embedded in a prominent myxoid stroma. In hypocellular or reticular areas, neoplastic cells were more spindle like and arranged in interweaving strands or cords forming a lace-like pattern	Mitoses were very rare (< 1/10 HPF)	Jeon et al. 2014)
30	49	F	No	EBC-	RLL	4	Well circumscribed, multinodular, and distinctly gelatinous, consisted of vague coalescent nodules composed of short spindle to ovoid or stellate cells embedded in a prominent myxoid stroma. In hypocellular or reticular areas, neoplastic cells were more spindle like and arranged in interweaving strands or cords forming a lace-like pattern. Moderate atypism with large hyperchromatic nuclei and nucleoli	Mitoses were very rare (< 1/10 HPF)	Jeon et al. (2014)
31	54	F	No	EBC +	RLL	4.5	Well circumscribed, multinodular, and distinctly gelatinous, consisted of vague coalescent nodules composed of short spindle to ovoid or stellate cells embedded in a prominent myxoid stroma. In hypocellular or reticular areas, neoplastic cells were more spindle like and arranged in interweaving strands or cords forming a lace-like pattern. There were many lymphoid cell and plasma cell infiltrations at the periphery or within the mass	Mitoses were very rare (< 1/10 HPF)	Jeon et al. (2014)
32	65	M	No	EBC +	LLL	13	Well circumscribed, multinodular, and distinctly gelatinous, consisted of vague coalescent nodules composed of short spindle to ovoid or stellate cells embedded in a prominent myxoid stroma. In hypocellular or reticular areas, neoplastic cells were more spindle like and arranged in interweaving strands or cords forming a lace-like pattern. Exhibited extensive myxoid changes and was infiltrated by many plasma cells and histiocytes throughout the entire lesion with scarce presence of neoplastic cells	Mitoses were very rare (< 1/10 HPF)	Jeon et al. (2014)
33	29	F	NM	EBC-	L	3	The tumor was composed of short spindle‐shaped to ovoid and stellate cells embedded in the reticular network of a myxoid stroma. Lymphoplasmacytic cell infiltration was also evident. The extent of cellular atypia was mild	Rare mitotic figures	Kim et al. (2017)
34	32	F	No	NM	RUL	3.5	Lobulated neoplasm composed of interanastomosing cords and small nests of predominantly epithelioid cells admixed with stellate-shaped cells suspended in a chondromyxoid matrix. Rare areas of hyalinisation were present. Microscopic foci of hemorrhage and necrosis were present	Rare mitotic figures	Yanagida et al. (2017)
35	48	M	Yes	NM	L	> 14	Low to moderate cellular neoplasm composed of bland looking medium-sized oval to rounded epithelioid cells arranged in a prominent reticular and microcystic lace-like chordoid pattern in a highly myxoid stroma. There was prominent vascularisation of the stroma	Mitoses were scant (< 2/10 HPF)	Agaimy et al. (2017)
36	41	F	NM	NM	R	5.1	Well-circumscribed tumor composed of a population of spindle-shaped, focally ovoid tumor cells growing in cords and fascicles embedded in a myxoid stroma, which stained positive with Alcian blue	The mitotic activity was brisk (> 11 /10 HPF)	Koelsche et al. (2020)
37	21	F	NM	EBC-	NM	NM	Abundant hypovascular, myxoid matrix was seen with focal hemosiderin deposition and a heterogeneous proliferation of middle-sized tumor cells, forming trabecular networks as well as rare solid areas. Oval nuclei and a variable amount of eosinophilic cytoplasm. In selected solid areas, a more pronounced nuclear atypia was seen	Lack of necrosis or mitotic activity	Opitz et al. (2019)
38	67	M	NM	NM	RLL	1.4	Well-circumscribed, lacked fibrous pseudocapsule and had an infiltrative border, consisted of reticular proliferation of short spindle to oval cells within an abundant myxoid stroma. Displayed mild nuclear atypia. A small number of lymphocytes and plasma cells were scattered within the tumor, and there was no peripheral lymphoid cuff	Mitotic figures were few	Nishimura et al. (2023)
39	80	F	NM	EBC +	LLL	NM	Fragments of a multinodular and variably cellular tumor predominantly composed of both slender and plump spindle cells on a prominently myxoid background The tumor stroma consisted of an abundant bluish myxoid matrix that was diffusively positive with Alcian blue and was sensitive to digestion by hyaluronidase	The mitotic activity was low (> 3/10 HPF)	Prieto-Granada et al. (2017)

*NM* Not mentioned, *NS* No symptom, *EBC* +*/− *endobronchial component involved (+) or not involved (−), *RUL* Right upper lobe, *RLL* Right lower lobe, *LLL* Left lower lobe, *LUL* Left upper lobe, *L* Left lung, *R* Right lung, *NED* no evidence of disease

### Imaging characteristics

Currently, CT scans serve as the primary imaging modality for PPMS diagnosis, with X-rays providing supplementary assistance. However, the specific imaging features of this tumor remain somewhat uncertain. Existing literature reviews and pathology reports on PPMS emphasize the significance of CT findings in enhancing our understanding of the disease. On CT scans, PPMS is frequently found in close proximity to the bronchi and often infiltrates the lung parenchyma. Most PPMS cases observed in imaging reports indicate that the primary lung masses are predominantly located in the right lower lobe (28%, 11/39) and right upper lobe (23%, 9/39). These tumors typically appear as well-defined solid masses with sizes ranging from 1.4 cm to 14 cm. Contrast-enhanced CT scans typically reveal these lesions as mildly and heterogeneously enhanced masses (Yuliana and Hayati 2022), with contrast enhancement in both solid and cystic components. Additionally, X-ray images of PPMS exhibit a lung field mass shadow, which may resemble a pleural effusion (Yuliana and Hayati 2022).

### Histopathologic characteristics

**Fig. 1:**
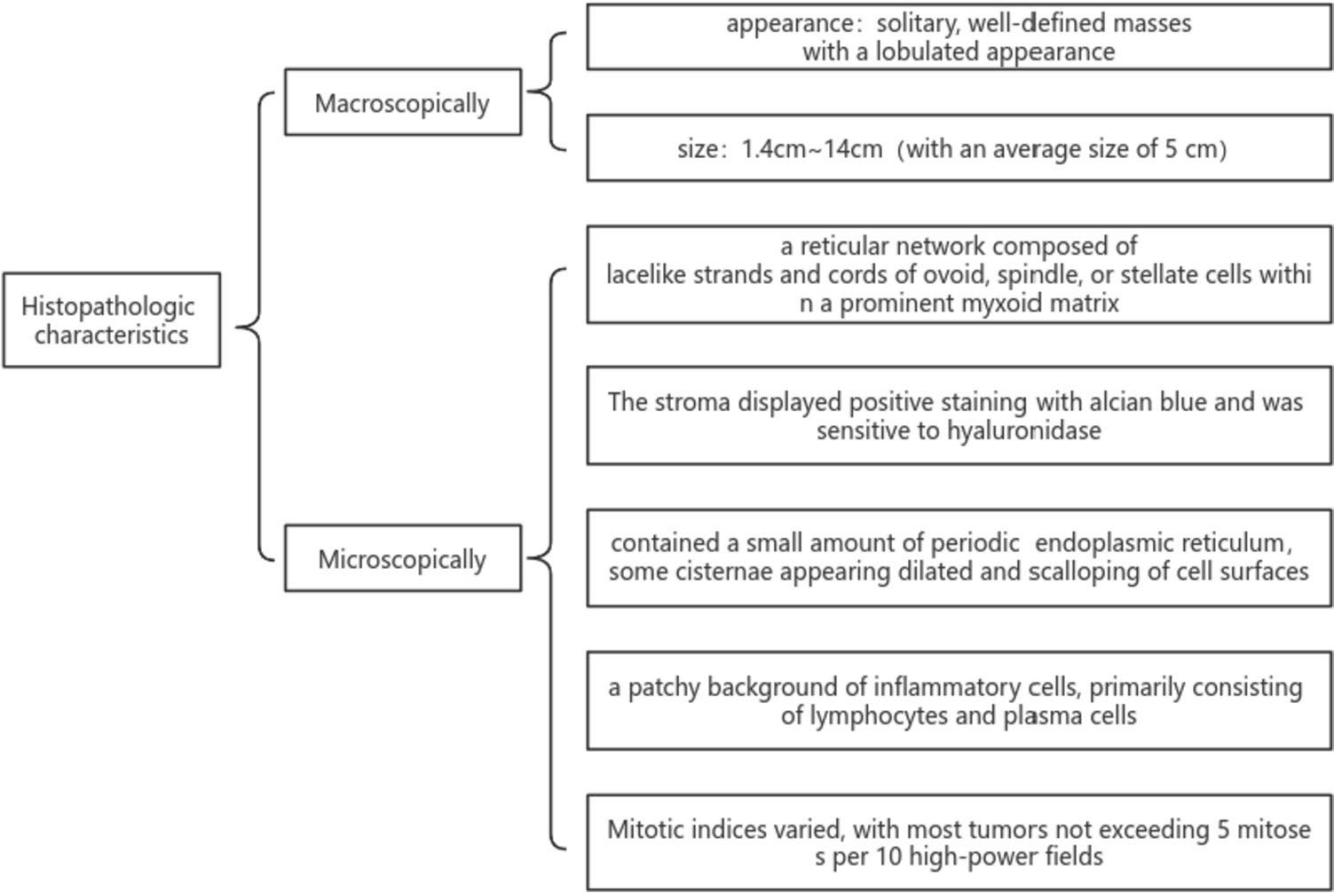
Histopathologic characteristics of PPMS with EWSR1::CREB1 fusion

Macroscopically, PPMS lesions typically manifest as solitary, well-defined masses with a lobulated appearance and a pale, crystalline cut surface. These tumors vary in size, ranging from 1.4 cm to 14 cm, with an average size of 5 cm. Microscopically, most cases exhibit a lobulated architecture, featuring a reticular network composed of delicate, lace-like strands and cords of ovoid, spindle, or stellate cells within a prominent myxoid matrix. Thway et al. (2011) reported clinicopathological data on 10 PPMS cases, which displayed cords of polygonal, spindle, or stellate cells embedded within a myxoid stroma, resembling extraskeletal myxoid chondrosarcoma. Two cases described by Nicholson et al. (1999) demonstrated interweaving cords of small, uniform, rounded, or slightly elongated cells within a myxoid stroma. The stroma displayed positive staining with Alcian blue and was sensitive to hyaluronidase. Tumor cells contained a small amount of periodic acid-Schiff-positive eosinophilic cytoplasm. Ultrastructural studies revealed an excess of rough endoplasmic reticulum in tumor cells, with some cisternae appearing dilated and scalloping of cell surfaces, although no intracisternal tubules were identified (Nicholson et al. 1999). In most cases, a patchy background of inflammatory cells, primarily consisting of lymphocytes and plasma cells, is present, contributing to the tumor’s infiltrative appearance and occasional focal necrosis and inflammation. Additionally, Chen et al. (2020) expanded the morphological and cytological spectrum of PPMS. They documented an additional case of this rare tumor and reported the first occurrence of chondrocyte-like and physaliferous-like tumor cells within this tumor, with a more intense inflammatory infiltrate in their case. Furthermore, PPMS often exhibits mild to moderate atypia; in the study by Thway et al. (2011), four cases displayed no or minimal atypia, six showed focal pleomorphism, and five had necrosis. Mitotic indices varied, with most tumors not exceeding 5 mitoses per 10 high-power fields (Fig. 1).

### Molecular genetics and immunohistochemistry

Recent studies have confirmed that the critical genetic feature of PPMS is the rearrangement of the *EWSR1* gene and the formation of the *EWSR1::CREB1* fusion gene. This genetic alteration serves as a crucial diagnostic marker. To identify the *EWSR1* gene rearrangement, fluorescence in situ hybridization (FISH) can be conducted on formalin-fixed paraffin-embedded (FFPE) tissue samples. This test significantly enhances diagnostic accuracy. In Table 2 (Nicholson et al. 1999; Wu et al. 2021; Thway et al. 2011; Matsukuma et al. 2012; Chen et al. 2020; Inayama et al. 2001; Smith et al. 2014; Jeon et al. 2014; Kim et al. 2017; Yanagida et al. 2017; Agaimy et al. 2017; Koelsche et al. 2020; Opitz et al. 2019; Nishimura et al. 2023; Prieto-Granada et al. 2017), which compiles the 39 PPMS cases, FISH analysis was performed in 34 cases, revealing a positive *EWSR1* gene rearrangement rate of 85% (29/34). Furthermore, the presence of the *EWSR1::CREB1* fusion can be demonstrated through reverse transcription-polymerase chain reaction (RT-PCR) targeting specific fusion transcripts. *EWSR1::CREB1* fusion is commonly found in various mesenchymal tumors occurring at different sites, displaying a broad spectrum of biological behaviors (Bale et al. 2018). Of the 26 cases in which RT-PCR was performed, the positive rate of *EWSR1::CREB1* fusion is 73% (19/26). *EWSR1::CREB1* fusion is an important characteristic of PPMS; however, it is essential to note that this fusion is not exclusive to PPMS but is also observed in other neoplasms, including angiomatoid fibrous histiocytoma (AFH), clear cell sarcoma, hyalinising clear cell carcinoma of the salivary gland, and clear cell carcinoma of the soft tissue or gastrointestinal tract (Thway and Fisher 2012; Cazzato et al. 2022; Stockman et al. 2012; Rossi et al. 2007). Additionally, Nishimura T et al. (Nishimura et al. 2023) reported a unique case of PPMS, harboring an *EWSR1::ATF1* gene fusion. Both *ATF1* and *CREB1*, belonging to the *CREB* family, can create fusion genes with *EWSR1* (Kao et al. 2017). If an *EWSR1::ATF1* fusion is detected in pulmonary myxoid sarcoma with *EWSR1::CREB1* translocation, the tumor may be classified as “pulmonary myxoid sarcoma with *EWSR1::CREB* translocation” (Hashimoto et al. 2019).

**Table 2:** Reported cases of primary pulmonary myxoid sarcoma (PPMS)

No	IHC	FISH result	RT-PCR result	Treatment	Follow-up (years)	References
1	CK-, S100-, desmin-, SMA-, CD34-	NM	NM	Surgery	NED/3	Nicholson et al. (1999)
2	CK-, S100-, desmin-, SMA-, CD34-	NM	NM	Surgery	NED/0.5	Nicholson et al. (1999)
3	NM	*EWSR1* gene rearrangement	NM	Surgery	NED/0.5	Wu et al. (2021)
4	NM	*EWSR1* gene rearrangement	NM	Surgery	Metastases to pleural and bone	Wu et al. (2021)
5	NM	*EWSR1* gene rearrangement	NM	Surgery	NED/2.4	Wu et al. (2021)
6	NM	*EWSR1* gene rearrangement	NM	Surgery	NED/1.9	Wu et al. (2021)
7	NM	*EWSR1* gene rearrangement	NM	Surgery	NED/2.4	Wu et al. (2021)
8	NM	*EWSR1* gene rearrangement	NM	Surgery	NED/2	Wu et al. (2021)
9	NM	*EWSR1* gene rearrangement	NM	Surgery	NED/0.3	Wu et al. (2021)
10	NM	NM	*EWSR1::CREB1* fusion	Surgery	NED/0.1	Wu et al. (2021)
11	NM	*EWSR1* gene rearrangement	NM	Surgery	NED/0.25	Wu et al. (2021)
12	NM	*EWSR1* gene rearrangement	NM	Surgery	NED/1	Wu et al. (2021)
13	CK-, S100-, desmin-	*EWSR1* gene rearrangement	*EWSR1::CREB1* fusion	Surgery	NED/15	Thway et al. (2011)
14	CK-, S100-, desmin-	*EWSR1* gene rearrangement	*EWSR1::CREB1* fusion	Surgery	NED/12	Thway et al. (2011)
15	S100 focal +, CK-, desmin-, p63-	*EWSR1* gene rearrangement	Neg	Surgery	NED/1	Thway et al. (2011)
16	CK-, EMA-, TTF-1-, S100-, desmin-	Neg	Neg	Surgery	Death followed a few months after the brain metastases	Thway et al. (2011)
17	CK-, EMA-,S100-, desmin-	*EWSR1* gene rearrangement	*EWSR1::CREB1* fusion	Surgery	NM	Thway et al. (2011)
18	EMA weak +, CK-, TTF-1-, S100-, HMB45-, melan A-, desmin-	Neg	*EWSR1::CREB1* fusion	Surgery	Left renal metastasis	Thway et al. (2011)
19	EMA weak +, CK-, TTF-1-, S100-, desmin-	*EWSR1* gene rearrangement	*EWSR1::CREB1* fusion	Surgery	NM	Thway et al. (2011)
20	EMA weak +, CK-, p63-, TTF-1-, S100-, desmin-	Neg	Neg	Surgery	NM	Thway et al. (2011)
21	EMA weak +, CK-, TTF-1-, S100-, HMB45-, melan A-, desmin-	*EWSR1* gene rearrangement	*EWSR1::CREB1* fusion	Surgery	NED/4	Thway et al. (2011)
22	NM	*EWSR1* gene rearrangement	*EWSR1::CREB1* fusion	Surgery	NM	Thway et al. (2011)
23	EMA focal +, CK-,TTF-1-, napsin A-, S100-, CD34-, desmin-, SMA-, CD10-, p63-, calponin-, caldesmon-, c-kit-,HMB-45-, synaptophysin-, GFAP-	NM	*EWSR1::CREB1* fusion	Surgery	NED/5.8	Matsukuma et al. (2012)
24	EMA +, CK-, TTF-1-, CAM5.2-, S100-, calponin-, SMA-, desmin-, *ALK*-, CD31-, CD34-	*EWSR1* gene rearrangement	*EWSR1::CREB1* fusion	Surgery	NED/3.1	Chen et al. (2020)
25	NM	NM	NM	Surgery	NED/3.8	Inayama et al. (2001)
26	EMA focal +, CK-, p63-, S100-, desmin-	*EWSR1* gene rearrangement	*EWSR1::CREB1* fusion	Surgery	NED/1.5	Smith et al. (2014)
27	Desmin +, EMA focal +, CK-, p63-, S100-	*EWSR1* gene rearrangement	Neg	Surgery	NED/1.3	Smith et al. (2014)
28	EMA focal +, CK-, p63-, S100-	Neg	Neg	Surgery	NED/0.3	Smith et al. (2014)
29	EMA focal +, CD99 focal weak +, SMA-, desmin-, caldesmon-, calponin-, S100-, CK-, CD31-, CD34-, p63-, CD56-, synaptophysin-	*EWSR1* gene rearrangement	*EWSR1::CREB1* fusion	Surgery	NED/0.7	Jeon et al. (2014)
30	EMA focal +, CD99 focal weak +, SMA focal +, desmin-, caldesmon-, calponin-, S100-, CK-, CD31-, CD34-, p63-, CD56-, synaptophysin-	*EWSR1* gene rearrangement	*EWSR1::CREB1* fusion	Surgery	NED/9.7	Jeon et al. (2014)
31	EMA focal +, CD99 focal +, SMA-, desmin-, caldesmon-, calponin-, S100-, CK-, CD31-, CD34-, p63-, CD56-, synaptophysin-	*EWSR1* gene rearrangement	*EWSR1::CREB1* fusion	Surgery	NED/12.6	Jeon et al. (2014)
32	EMA focal +, CD99 focal +, SMA-, desmin-, caldesmon-, calponin-, S100-, CK-, CD31-, CD34-, p63-, CD56-, synaptophysin-	*EWSR1* gene rearrangement	*EWSR1::CREB1* fusion	Surgery	Metastases to contralateral lung	Jeon et al. (2014)
33	EMA +, SMA-, SMMHC-, calretinin-, TTF-1-, CK-, p63-, S100-, CD34-, CD56-	*EWSR1* gene rearrangement	*EWSR1::CREB1* fusion	Surgery	NED/1.4	Kim et al. (2017)
34	CD68 weak +, CD163 weak +, synaptophysin weak +, CK-, EMA-, calponin-, GFAP-, SMA-, desmin-, caldesmon-, S100-, HMB-45-, CD34-, CD31-, chromogranin-	*EWSR1* gene rearrangement	*EWSR1::CREB1* fusion	Surgery	NED/8	Yanagida et al. (2017)
35	CD10 focal +, EMA focal +, CK-, TTF-1-, ERG-, CD31-, p63-, desmin-, SMA-, S100-, CD34-, CD30-, MUC4-, TLE1-, STAT6-	Neg	Neg	Surgery	Metastases to Cerebellar	Agaimy et al. (2017)
36	NM	*EWSR1* gene rearrangement	*EWSR1::CREB1* fusion	Surgery	NED/0.9	Koelsche et al. (2020)
37	CK weak +, SMA +, INI1-, EMA-, S100-, desmin-, ERG-, MDM2-, CDK4-	*EWSR1* gene rearrangement	*EWSR1::CREB1* fusion	Surgery	NED/3.1	Opitz et al. (2019)
38	SMA +, EMA +, desmin-, CD31-, CD34-, STAT6-, calretinin-, HEG1-, D2-40-, S100 protein-, CEA-, broad keratin-, keratin5/6-, ALK-, TTF-1-	*EWSR1* gene rearrangement	*EWSR1::ATF1* fusion	Surgery	NED/1.0	Nishimura et al. (2023)
39	EMA focal +, CK-, S100-, HMB45-, CD31-, CD34-, SMA-, caldesmon-H-, desmin-, GFAP-	*EWSR1* gene rearrangement	NM	Surgery	NED/3	Prieto-Granada et al. (2017)

*IHC* immunohistochemistry*, Neg* negative to *EWSR1* gene rearrangement or *EWSR1::CREB1* fusion

PPMS lacks highly specific immunohistochemical markers. Immunohistochemical analysis has shown that tumor cells consistently express epithelial membrane antigen (EMA) (Wu et al. 2021). Conversely, they do not exhibit immunoreactivity for cytokeratin (CK), thyroid transcription factor-1 (TTF-1), napsin A, S-100 protein, CAM5.2, CD10, CD31, CD34, desmin, smooth-muscle actin (SMA), p63, calponin, h-caldesmon, anaplastic lymphoma kinase (*ALK*), c-kit, melanocytic markers (HMB-45), synaptophysin, or glial fibrillary acid protein (GFAP) (Chen et al. 2020).

The key genetic characteristics of PPMS involve *EWSR1* rearrangement and *EWSR1::CREB1* fusion. Additionally, immunohistochemistry typically reveals positivity for EMA. The combination of these genetic and immunohistochemical features aids in the diagnosis of this tumor.

### Diagnosis and differential diagnosis

PPMS lacks distinctive clinical manifestations, and routine blood tests and tumor marker levels typically fall within the normal range, making it challenging to differentiate from other lung conditions with similar clinical presentations. Therefore, the diagnosis of PPMS primarily relies on the tumor's location and histopathological features. Pathological diagnosis is typically obtained through needle biopsy, which serves as the gold standard for clinical diagnosis and holds significant importance for patient management. Differential diagnosis is crucial in the evaluation of PPMS. PPMS should be distinguished from several thoracic tumors:

#### Extraskeletal myxoid chondrosarcoma (EMC)

EMC is a rare tumor characterized by multinodular growth and short anastomotic strands of oval to spindle-shaped cells embedded in a rich myxoid matrix (Balanzá et al. 2016; Zhou et al. 2012). EMC possesses distinct ultrastructural features, with cords of cells immersed in a matrix rich in glycosaminoglycans (Goh et al. 2001). The key distinguishing factor between PPMS and EMC is their genetic differences. Genetically, gene fusions involving nuclear receptor subfamily 4 group A member 3 (*NR4A3*) and resulting in *NR4A3* constitutive expression are exclusive to EMC and considered a hallmark of the disease (Stacchiotti et al. 2020). The primary fusion genes identified in EMC are *EWSR1::NR4A3* and TATA-box binding protein-associated factor 15::nuclear receptor subfamily 4 group A member 3 (*TAF15::NR4A3*) gene fusions (Hisaoka and Hashimoto 2005), whereas PPMS is characterized by the *EWSR1::CREB1* gene fusion.

#### AFH

AFH is a rare soft tissue mesenchymal neoplasm (Thway and Fisher 2015). According to a study by Gui et al. (2020), PPMS overlaps with a myxoid variant of AFH of the soft tissue morphologically. The myxoid variant of AFH occurs mainly in soft tissues and is rarely seen in the lungs, myxoid variant AFH exhibits distinct myxoid features and can often positively express EMA and EWSR1 gene rearrangements. As a result, it is difficult to distinguish from PPMS (Gong et al. 2018). But, the myxoid stroma of pulmonary AFH is usually focal. The most common morphological features of AFH cases include a peritumoral lymphoid cuff and whorled or storiform patterns, which are not typically described in PPMS. Immunophenotype differences also serve as distinguishing factors; AFH often exhibits positivity for CD68, CD163, desmin, EMA, and *ALK* (Wang et al. 2021). In contrast, PPMS is characterized by positivity for EMA, along with negativity for desmin and *ALK*. Moreover, low-to-intermediate malignant potential AFH can also exhibit *EWSR1::CREB1* fusion due to t(2; 22) (q33; q12) (Antonescu et al. 2007; Costa and Weiss 1990). While *EWSR1::ATF1* and fused in sarcoma::activating transcription factor 1 (*FUS::ATF1*) fusion genes can be detected in AFH, they are rarely found in PPMS cases (Chen et al. 2020).

Gui et al. (2020) noted that PPMS and AFH have similar histological features, clinical manifestations, immunophenotypic and molecular alterations, and that they are closely related in lineage and pathogenesis. However, whether EWSR1-positive PPMS and AFH may represent the lineage of the same disease, and whether they originate from primitive mesenchymal cells driven by the same or similar EWSR1 fusion gene products, remains to be further investigated.

#### Myoepithelial tumors (MT)

Myoepithelial tumors (MT) exhibit diverse morphological characteristics and immunophenotypes. They typically display a multinodular or lobular growth pattern, consisting of spindled, ovoid, or epithelioid cells. Immunohistochemically, over 44% of MT cases show the presence of *EWSR1* gene rearrangement, and most MT cases test positive for CK, p63, S-100 protein, calponin, and SMA (Jo and Fletcher 2015), whereas these markers are negative in PPMS. Additionally, common fusion variants in MT include *EWSR1::POU5F1*, *EWSR1::PBX1,* and *EWSR1::ZNF444* (Wang et al. 2021), while the primary fusion gene in PPMS is *EWSR1::CREB1* fusion gene.

#### Inflammatory myofibroblastic tumor (IMT)

IMT is a distinct tumor characterized by myofibroblastic spindle cells and accompanied by an inflammatory infiltrate of plasma cells, lymphocytes, and eosinophils (Khatri et al. 2018). IMTs are immunohistochemically positive for SMA, *ALK-1*, desmin, and calponin (Henriques de Gouveia 2023). Approximately 50–70% of IMTs harbor the *ALK* gene rearrangement (Gilani and Kowalski 2014), while PPMS is negative for these markers.

#### Low-grade myxoid liposarcoma (LGML)

LGML is a malignant adipogenic neoplasm with prominent arborizing capillaries, occasional lipoblasts, and primitive spindle cells in a myxoid background. LGML often involves DNA damage-inducible transcript 3 (*DDIT3*) gene rearrangement (Scapa et al. 2021), which is not present in PPMS.

#### Pulmonary microcystic fibromyxoma (PMF)

**Fig. 2:**
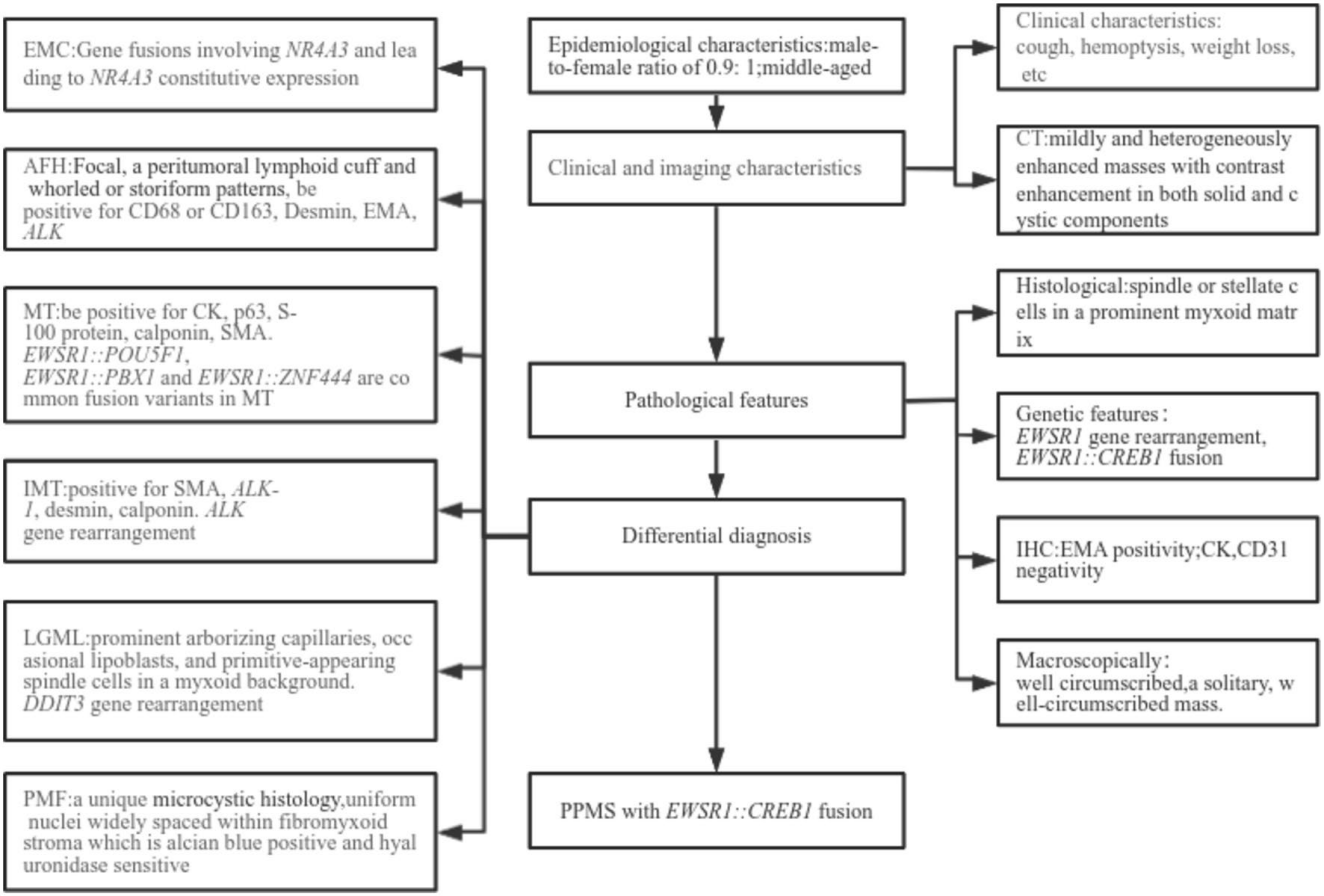
The diagnostic flow chart of PPMS with EWSR1::CREB1 fusion

PMF is well circumscribed with notable cystic changes and a myxoid stroma. Microscopically, PMF features innocuous, widely spaced, spindled to stellate tumor cells with minimal nuclear pleomorphism, and no mitotic activity. These uniform nuclei are widely dispersed within a fibromyxoid stroma, which stains positively with Alcian blue and is sensitive to hyaluronidase. PMF is distinct in its unique microcystic histology, which is absent in PPMS. PMF does not exhibit diagnostic molecular genetic changes, and it does not present with endobronchial localization (Shilo et al. 2006) (Fig. 2).

### Therapy strategies

Currently, the primary treatment for PPMS with *EWSR1::CREB1* fusion is surgery. Given the rarity and non-specific clinical features of PPMS, there are no definite factors known to impact its prognosis.

#### Surgery

Surgical excision is commonly performed as the primary treatment for all patients with PPMS (Wu et al. 2021). It is essential to closely monitor patients post-surgery to assess the effectiveness of the treatment and the prognosis. PPMS, being a well-defined, low-grade malignant solid sarcoma with a low Ki-67 index, has shown favorable clinical outcomes following surgical resection (Wu et al. 2021). According to existing literature, surgery is the primary treatment approach for most patients. The available surgical excision options include wedge resection, segment resection, lobectomy, and pneumonectomy, depending on tumor size and stage. For instance, Wu et al. (2021) reported a case where a patient with PPMS had primary thyroid cancer with lymph node and lung metastases. In this case, the patient underwent thoracoscopic right upper lobectomy and lymph node dissection, followed by bilateral total thyroidectomy and neck lymph node dissection three months later. There were no signs of recurrence or metastasis during the 12-month follow-up period. Additionally, Kim et al. (2017) described a case where the mass was located in the interlobar fissure of the left lung without definite parenchymal invasion. They successfully removed the mass via video-assisted thoracoscopic surgery without the need for pulmonary parenchymal resection. Thus, a comprehensive treatment approach, with surgery as the primary mode and active management of the primary disease, can improve the condition and quality of life for PPMS patients. Accurate TNM staging and molecular pathological classification can guide postoperative comprehensive treatment. In summary, surgical treatment is the mainstay for PPMS patients and can yield positive outcomes. For patients who are not suitable for surgical resection, alternative treatments such as medication may be considered.

#### Chemotherapy

Currently, there are limited data on chemotherapy for PPMS, and its effectiveness remains unclear, warranting further investigation. Garnier et al. (2021) initially reported a doxorubicin-based chemotherapy regimen used to treat intracranial non-myxoid angiomatoid fibrous histiocytoma with *EWSR1::CREB1* transcript fusion, which resulted in prolonged stable disease for fourteen months after treatment discontinuation. Although PPMS shares the *EWSR1::CREB1* fusion with AFH, there have been no reports indicating that doxorubicin can produce a significant therapeutic effect in PPMS with *EWSR1::CREB1* fusion. Consequently, the chemotherapy regimens for PPMS with *EWSR1::CREB1* fusion remain uncertain.

#### Molecularly targeted therapy

While EWSR1 gene rearrangement and *EWSR1::CREB1* fusion are distinctive features of PPMS, the current understanding of this target remains limited. Therefore, it is necessary to investigate the potential effectiveness of targeted therapy in treating PPMS. Subbiah et al. (2016) have explored the use of the cellular mesenchymal–epithelial transition factor (*c-Met*)/*ALK* inhibitor crizotinib and the multi-kinase vascular endothelial growth factor (VEGF) inhibitor pazopanib in metastatic gastrointestinal neuroectodermal tumor (GNET) with *EWSR1::CREB1* fusion. These two drugs demonstrated a sustained, nearly complete response in GNET. Additionally, the study included 11 cases of patients with the same *EWSR1::CREB1* fusion, encompassing sarcomas not otherwise specified (NOS), malignant neoplasms of unknown primary, melanoma, and head and neck mucoepidermoid carcinoma. *EWSR1::CREB1* fusion was identified as the primary driver in these cases. Subbiah et al. (2016) suggested that patients with this *EWSR1::CREB1* fusion could also benefit from crizotinib and pazopanib. Furthermore, Ngo et al. (2022) reported a durable response to crizotinib in metastatic angiomatoid fibrous histiocytoma with *EWSR1::CREB1* fusion and *ALK* overexpression. However, the use of crizotinib and pazopanib in clinical treatment for PPMS with *EWSR1::CREB1* fusion has not been studied yet. The effectiveness of these two drugs in PPMS cases with *EWSR1::CREB1* fusion remains uncertain, and further data are needed to determine their suitability for treating PPMS with *EWSR1::CREB1* fusion.

### Prognosis

Based on available research findings, the clinical stage of the tumor, the overall health of the patients, their age, and gender all play significant roles as prognostic factors. Retrospective studies indicate that most patients showed good recovery following surgery, with no evidence of recurrence or metastasis. According to Table 2, the rate of metastasis after surgical interventions for PPMS is 14.7% (5/34), with only one fatality resulting from brain metastases after surgery (Thway et al. 2011). Among the 34 patients who underwent surgery, 85.3% (29/34) survived at the final follow-up with NED. The NED duration for these 29 patients ranged from 0.1 to 15 years, with an average NED of 3.5 years. However, based on retrospective studies, there is no conclusive clinical evidence establishing a direct link between post-surgery metastasis and the presence of *EWSR1* gene rearrangement or *EWSR1::CREB1* fusion. The role of these genetic abnormalities in predicting the prognosis of PPMS remains uncertain and requires further investigation in the future.

## Conclusion

PPMS with *EWSR1::CREB1* fusion is an exceedingly rare low-grade malignant sarcoma, typically found in the bronchi and lung parenchyma. Microscopically, PPMS is characterized by a reticular network with delicate lace-like strands and cords of ovoid, spindle, or stellate cells in a prominent myxoid matrix. *EWSR1* rearrangement and *EWSR1::CREB1* fusion are crucial genetic features of PPMS and serve as important diagnostic markers. Immunohistochemically, PPMS tests positive for EMA. In terms of treatment, surgery has been the primary approach in recent years. Therefore, the efficacy of other treatments such as radiotherapy, chemotherapy, and immunotargeted therapy still requires further investigation.

## Data Availability

A comprehensive search was performed through PubMed using the literature retrieval strategy “[Primary Pulmonary myxoid sarcoma (Title/Abstract)] AND [*EWSR1::CREB1* fusion (Title/Abstract)]” in July 2023. Relevant articles were obtained, and references from each of these articles were further searched for relevant articles. A total of 46 articles were reviewed (15 were case reports or case series).
